# Drought, Extreme Heat, and Intimate Partner Violence in Low- and Middle-Income Countries

**DOI:** 10.1001/jamanetworkopen.2025.27818

**Published:** 2025-08-20

**Authors:** Pin Wang, Lingzhi Chu, Jie Ban, Ernest O. Asare, Muthusamy Sivakami, Alexandra Restrepo Henao, Lucy Chimoyi, Tami P. Sullivan, Kai Chen

**Affiliations:** 1 Department of Global, Environmental, and Occupational Health, School of Public Health, University of Maryland, College Park; 2 Department of Environmental Health Sciences, Yale School of Public Health, Yale University, New Haven, Connecticut; 3 Yale Center on Climate Change and Health, Yale School of Public Health, Yale University, New Haven, Connecticut; 4 Department of Epidemiology of Microbial Diseases, Yale School of Public Health, New Haven, Connecticut; 5 School of Health Systems Studies, Tata Institute of Social Sciences, Mumbai, India; 6 Epidemiology Group, National School of Public Health, University of Antioquia, Medellín, Colombia; 7 Implementation Research Division, The Aurum Institute, Johannesburg, South Africa; 8 Department of Psychiatry, Yale University School of Medicine, New Haven, Connecticut

## Abstract

**Importance:**

Recent evidence has shown that elevated temperatures are associated with a higher risk of intimate partner violence (IPV), a serious violation of human rights. However, it remains unclear whether drought also heightens the risk and whether drought and extreme heat jointly affect IPV.

**Objective:**

To investigate the interactive association between drought, extreme heat, and IPV in 42 low- and middle-income countries (LMICs).

**Design, Setting, and Participants:**

This cross-sectional study included women answering multicluster surveys in sub-Saharan Africa, South and Southeast Asia, Latin America, and the Caribbean between 2003 and 2020. The analysis was conducted from January to July 2024.

**Exposures:**

Drought was measured by the standardized precipitation evapotranspiration index at a timescale of 1, 3, 6, or 12 months. Extreme heat days were defined as days with maximum temperature above the 90th, 92.5th, 95th, or 97.5th percentile of the cluster-specific distribution. The respective numbers of extreme heat days and drought months were calculated to evaluate the interaction between drought and extreme heat.

**Main Outcomes and Measures:**

IPV against women experienced in the past 12 months before the survey, including emotional, physical, and sexual violence.

**Results:**

Among a total of 494 471 women (mean [SD] age, 32.1 [8.5] years) surveyed, the prevalence for any, emotional, physical, and sexual IPV was 28.3% (139 901 women), 16.8% (83 100 women), 19.5% (96 531 women), and 7.4% (36 804 women), respectively. Overall drought exposures at all timescales were associated with increased IPV risk, with the largest effect size at the 12-month scale (relative risk [RR], 1.07 [95% CI, 1.06-1.09]), where mild (RR, 1.08 [95% CI, 1.06-1.09]) and severe (RR, 1.06 [95% CI, 1.04-1.08]) drought were also associated with any IPV. When stratifying violence IPV by type, the largest effect sizes were observed for overall drought with emotional violence at the 1-month scale and with physical violence at the 12-month scale. When estimating relative excess risk due to interaction in 2 ways, there was a negative and significant interaction between drought and extreme heat, defined using the threshold of the 97.5th percentile of the distribution.

**Conclusions and Relevance:**

In this cross-sectional study of 42 LMICs, drought conditions with various durations were associated with increased IPV risk. Given the increasing extreme events under climate change, there is a pressing need for enhanced initiatives to prevent domestic violence.

## Key Points

**Question:**

Are drought conditions or drought with extreme heat conditions associated with an increased risk of intimate partner violence (IPV) in low- and middle-income countries?

**Findings:**

In this cross-sectional study using global survey data for 494 471 women, 12-month drought was associated with an estimated 7% increase in IPV risk. We found a negative and significant interaction between drought and extreme heat.

**Meaning:**

These findings underscore the major global health implications of pressing climate change–related threats to women’s health, and thus the urgency to implement actionable interventions and climate adaptation measures to achieve gender equality.

## Teaser

This cross-sectional study of survey data from women in low- and middle-income countries examines the association of drought and extreme heat with intimate partner violence.

## Introduction

Intimate partner violence (IPV) against women, characterized as violence perpetrated by a husband or male intimate partner, is one of the most common forms of gender-based violence, hindering the achievement of gender equality through United Nations’ Sustainable Development Goal 5.2 (ie, eliminating all forms of violence against all women and girls in public and private spheres).^1,2^ IPV poses a significant global public health risk, infringing upon human rights and causing short-term and long-term physical and mental health consequences, such as injuries, sexually transmitted infections, substance use, depression, and even suicide and homicide death.^2,3,4,5^ Estimates of the global lifetime IPV prevalence among women aged 15 years and above in 2013^2^ and 2022^5^ showed a subtle decrease from 30% in 2010 to 27% in 2018, with the highest prevalence in low- and middle-income countries (LMICs) in Oceania, central sub-Saharan Africa, Andean Latin America, and South Asia.^2,5^

Human society has been experiencing the immense impact of climate change and its induced extreme events with unprecedented frequency and intensity. For decades, scientists have been seeking to uncover the potential association between unfavorable climatic conditions, especially hot weather, and interpersonal violence.^6,7,8^ In particular, evidence in studies from 2020 and 2023^9,10,11^ has shown that higher temperatures increased the IPV risk in both high- and low-income settings. For example, a 2023 study^11^ found that every 1 °C increase in the annual mean temperature was associated with a 4.5% increase in the risk in 3 South Asian countries.

Extreme events can heighten the risk of gender-based violence through multiple mechanisms, including deteriorating housing conditions, food insecurity, financial hardship, postdisaster trauma, gender and cultural norms, law enforcement misconduct, the absence of privacy during displacement, health care and social service suspension, and civil wars.^12,13,14^ However, limited epidemiological evidence exists.^12,15^ Despite all using the Demographic and Health Surveys (DHS)^16^ across LMICs, inconsistent findings have been reported on the associations between dry conditions and IPV, including either significantly positive^17,18,19^ or nonsignificant^20,21,22^ associations in sub-Saharan Africa,^17,20,21^ India,^18,22^ and Peru.^19^ Except for 1 study with no specification,^22^ most of the current research used negative rainfall shocks as the indicator of dry conditions,^17,18,19,20,21^ which does not take into account evapotranspiration that transfer water content from the land surface to the atmosphere via evaporation and transpiration. In addition, there has been more evidence on temperature exposure,^9,10,11^ as opposed to extreme heat events. We only identified 1 study in Spain demonstrating increased risks of femicides, police reports, and helpline calls due to IPV associated with heat extremes over 34 °C.^23^

With low precipitation as the primary force, drought is a multifaceted environmental phenomenon shaped by various climatological factors influencing the balance between water supply and demand.^24^ Among them, temperatures play an imperative role in evapotranspiration, making it an indispensable parameter when calculating drought.^24^ Current evidence solely dependent on limited precipitation suggests a necessity for studies on more complex drought measures. Moreover, high temperatures may further exacerbate drought conditions by intensifying evapotranspiration. However, evidence remains lacking regarding the combined impact of extreme heat and drought on IPV. Accordingly, this study aimed to (1) examine the association between drought at various timescales and IPV in LMICs and (2) explore the potential joint association of drought and extreme heat on IPV.

## Methods

Analysis was conducted from January to July 2024 following the Strengthening the Reporting of Observational Studies in Epidemiology (STROBE) reporting guideline for cross-sectional studies. The Yale institutional review board determined this study as not human participant research; thus, ethics approval for this study was not required.

### Violence and Environmental Data

We extracted IPV occurrences from 2003 through 2020 from the DHS program, a nationally representative household survey system conducted in over 90 LMICs worldwide since 1984.^16^ DHS adopts a 2-stage sampling approach: (1) selecting survey clusters stratified by geographic region and urban or rural residence; and (2) within each cluster, 20 to 30 households are randomly sampled for interviews.^25^ Each woman was queried about whether she had experienced violence from her husband or household partner in the past 12 months before the survey, including emotional, physical, and sexual violence (eTable 1 in Supplement 1). In the analysis, we used a binary indicator for any IPV (emotional, physical, and sexual IPV combined) or 1 of these subtypes. To allow for comparison across surveys and countries, we computed a wealth index from multiple household attributes using a principal component analysis, as described in our previous work.^26^

We measured drought by the standardized precipitation evapotranspiration index (SPEI), which describes the difference between water supply, measured by precipitation, and water demand, measured by potential evapotranspiration.^24^ Water demand was calculated using a series of monthly gridded meteorological variables at a resolution of 0.1° (approximately 9 km), including maximum and minimum temperature, dewpoint temperature, wind speed, incoming solar radiation, and air pressure, obtained from the ERA5-Land dataset.^27^ We calculated the SPEI at timescales of 1, 3, 6, and 12 months (hereafter, SPEI-1, SPEI-3, SPEI-6, and SPEI-12) to capture both the short-term and long-term impact of meteorological conditions on drought status. It is important to note that the derived SPEI is a monthly metric representing the cumulative impacts of climatic conditions over a previous period. For instance, we calculated SPEI-12 for a given month by integrating the climatic variables for that month and 11 previous months to sum the water balance during the entire 12 months. We described the detailed calculation methods elsewhere.^26^ We defined drought severity according to the classification method by the Federal Office of Meteorology and Climatology MeteoSwiss (overall drought, SPEI ≤ −0.5; mild drought: −1.3 < SPEI ≤ −0.5; severe drought, SPEI ≤ −1.3).^26^

We then linked the gridded SPEI from 2002 through 2019 with each participant based on the geographic location of survey clusters and the interview date from 2003 through 2020. Because the reporting window from the woman spanned 12 months, making the exact date of IPV occurrence unknown, we used the SPEI in the 12th month before the survey to ensure that IPV occurred after potential drought exposure.

The extreme heat indicator was represented by the total number of extreme heat days during previous months (ie, 1, 3, 6, or 12) using thresholds of the 90th, 92.5th, 95th, or 97.5th percentile of the survey cluster–specific maximum temperature distribution between 2002 and 2019. Similar to drought assessment, we used the 12th month before the survey as the ending month for extreme heat assessment to ensure temporality. For example, we calculated the total number of 12-month extreme heat days spanning from 23 to 12 months before the survey. The calculation timeframes for drought and extreme heat indicators are illustrated in eFigure 1 in Supplement 1.

### Statistical Analysis

We employed log-binomial generalized linear mixed models by regressing the binary indicator for IPV on the categorical drought indicator, with nested random intercepts for country and survey cluster, allowing for variations across countries and clusters. Log-binomial regression was selected because the outcome was common. The covariates in the models included partner’s age and education, area of residence, and quintiles of the computed wealth index. Natural cubic splines of average mean temperature and survey year with 3 degrees of freedom were also included to adjust for the association with temperature and long-term trend. The average mean temperature was calculated during the same calculation period for the SPEI. For example, for the SPEI-12 model, we included the average temperature between the 23rd and 12th months before the survey. We then estimated the association with any IPV by drought severity and the association with overall drought (mild and severe drought combined) by IPV type.

Next, we examined the potential lag of the association of drought at various timescales with any IPV, employing log-binomial generalized additive models together with distributed lag nonlinear models.^28^ We used 11 months as the maximum lag period, with linear and natural spline functions with 3 degrees of freedom for the predictor and lag matrices, respectively. The detailed methods for the lagged association are described in eAppendix 1 in Supplement 1.

We examined potential effect modification by incorporating an interaction term between drought and each of the following variables: woman’s age (median age under 31 years; 31 years or older), her partner’s age (median age under 37 years; 37 years and older), education for both the respondent and her partner (primary and below; secondary and above), area of residence (urban; rural), wealth index (first and second quintile; third, fourth, and fifth quintile), if the partner drinks alcohol (never; ever), if the partner gets drunk (never; ever), and if the woman and her partner worked in the past 12 months (no; yes). We used the *P* value of the interaction term to assess the statistical significance of subgroup differences.

We applied 2 methods to explore the interactive association of drought and extreme heat with IPV during the 12-month period from 23 to 12 months before the interview. First, we separately calculated the total number of drought months at various timescales and the total number of extreme heat days above various thresholds and tested their individual associations with any IPV. Then we included an interaction term in the model and calculated the additive interaction represented by the relative excess risk due to interaction (RERI). Second, we computed the total number of extreme heat days in drought months, extreme heat days in nondrought months, non–extreme heat days in drought months, and non–extreme heat days in nondrought months during the same period. Due to the perfect collinearity of these 4 metrics (their sum equals 12 months), we included the first 3 metrics in the model, where non–extreme heat days in nondrought months served as a reference for the other 3 metrics. We then calculated RERI using coefficients from the model. The detailed calculation method is described in eAppendix 2 in Supplement 1.

We tested model robustness using multiple sensitivity analyses (eAppendix 3 in Supplement 1). All data analyses were completed using R statistical software version 4.3.1 (R Project for Statistical Computing). All results are reported as the relative risk (RR) associated with exposure to drought and/or extreme heat compared with nonexposure to drought and/or extreme heat, along with 95% CIs. A 2-sided *P* < .05 was considered significant.

## Results

**Figure 1.**
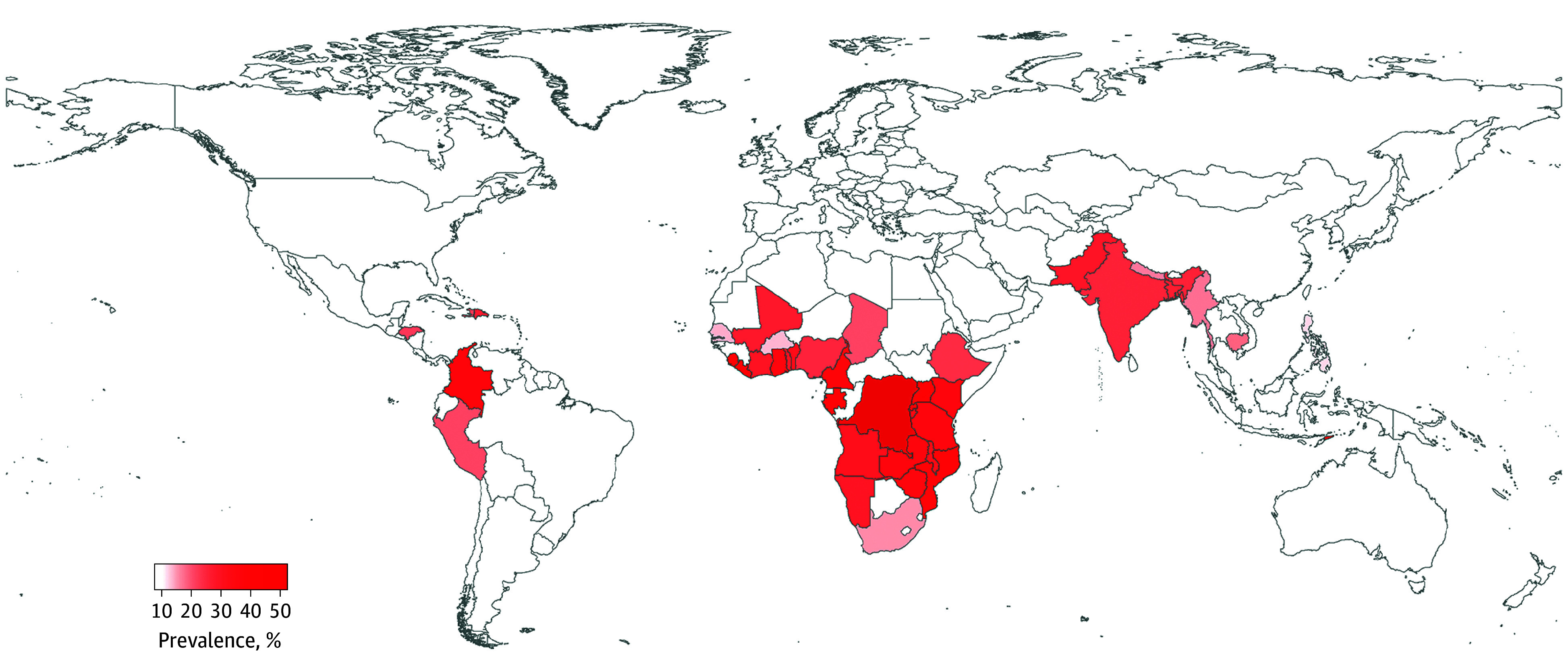
Prevalence of Intimate Partner Violence Against Women During the Past 12 Months Before the Survey, 2003-2020

A total of 494 471 women from 73 surveys in 42 countries were included in our analysis (age below 31 years at time of survey response, 233 451 [47.2%]) (eTable 2 in Supplement 1). The overall prevalence for any IPV across all participants was 28.3% (139 901 women) (Table), and the prevalence for emotional, physical, and sexual IPV was 16.8% (83 100 women), 19.5% (96 531 women), and 7.4% (36 804 women), respectively. Democratic Republic of the Congo exhibits the highest prevalence of any (4460 of 8540 [52.2%]), physical (3300 of 8540 [38.6%]), and sexual (2021 of 8540 [23.7%]) IPV, whereas Liberia has the highest prevalence of emotional IPV (2151 of 6244 [34.4%]) (Figure 1; eFigure 2 in Supplement 1). Democratic Republic of the Congo also experienced the greatest drought events at all timescales during the study period, whereas the Philippines experienced the least (eFigure 3 in Supplement 1). With the increase in timescale, there was an increase in the variance of the number of drought events experienced across all studied regions (eFigure 4 in Supplement 1). The number of extreme heat days decreased with the increase in extreme heat intensity, and the number of women exposed to drought or extreme heat decreased with the increase in drought timescale or extreme heat intensity, respectively (eTables 3 and 4 in Supplement 1). During the 23rd to 12th months before the survey, more women experienced both overall drought and extreme heat than a single type of event (eTables 5 and 6 in Supplement 1).

**Table.** Study Population Characteristics

Characteristic	Individuals, No. (%) (N = 494 471)					Intimate partner violence prevalence (%)^b^
	Missing	SPEI-12 drought exposure in 12 mos before survey^a^		Intimate partner violence occurrence		
		Yes	No	Yes	No	
Total	0	155 351	311 599	139 901	354 570	28.3
Respondent age, y						
<31	0	76 387 (49.2)	145 669 (46.7)	70 125 (50.1)	163 326 (46.1)	30.0
≥31		78 964 (50.8)	165 930 (53.3)	69 776 (49.9)	191 244 (53.9)	26.7
Partner age, y						
<37	56 931 (11.5)	65 949 (47.0)	134 236 (49.1)	61 997 (50.6)	149 197 (47.4)	29.4
≥37		74 501 (53.0)	138 927 (50.9)	60 536 (49.4)	165 810 (52.6)	26.7
Respondent education						
No education	25 (<0.1)	52 691 (33.9)	80 919 (26.0)	38 424 (27.5)	99 318 (28.0)	27.9
Primary		47 151 (30.4)	105 998 (34.0)	52 375 (37.4)	108 700 (30.7)	32.5
Secondary		43 604 (28.1)	98 192 (31.5)	41 579 (29.7)	110 995 (31.3)	27.3
Higher		11 898 (7.7)	26 473 (8.5)	7514 (5.4)	35 541 (10.0)	17.5
Partner education						
No education	24 480 (5.0)	40 741 (27.7)	58 338 (19.7)	27 985 (20.9)	74 318 (22.1)	27.4
Primary		40 216 (27.3)	94 432 (31.8)	46 226 (34.5)	95 184 (28.3)	32.7
Secondary		49 681 (33.8)	112 694 (38.0)	49 434 (36.9)	124 346 (37.0)	28.4
Higher		16 468 (11.2)	31 179 (10.5)	10 153 (7.6)	42 345 (12.6)	19.3
Residence						
Urban	0	53 722 (34.6)	120 996 (38.8)	52 430 (37.5)	138 005 (38.9)	27.5
Rural		101 629 (65.4)	190 603 (61.2)	87 471 (62.5)	216 565 (61.1)	28.8
Wealth quintile						
First (lowest)	38 125 (7.7)	35 191 (23.7)	53 737 (19.0)	31 565 (24.1)	60 291 (18.5)	34.4
Second		30 359 (20.5)	56 533 (20.0)	28 578 (21.8)	61 430 (18.9)	31.8
Middle		31 079 (21.0)	56 822 (20.1)	26 376 (20.2)	65 887 (20.2)	28.6
Fourth		29 616 (20.0)	55 100 (19.5)	23 515 (18.0)	67 364 (20.7)	25.9
Fifth (highest)		21 960 (14.8)	61 031 (21.5)	20 788 (15.9)	70 552 (21.7)	22.8
Partner alcohol drinking						
Never	9779 (2.0)	94 002 (61.9)	172 870 (56.6)	60 683 (44.5)	218 780 (62.8)	21.7
Ever		57 817 (38.1)	132 629 (43.4)	75 744 (55.5)	129 485 (37.2)	36.9
Respondent worked last year						
No	99 (<0.1)	53 235 (34.3)	116 692 (37.5)	43 778 (31.3)	135 556 (38.2)	24.4
Yes		102 062 (65.7)	194 866 (62.5)	96 090 (68.7)	218 948 (61.8)	30.5
Partner worked last year						
No	20 683 (4.2)	2720 (1.8)	5975 (2.0)	2687 (2.0)	6456 (1.9)	29.4
Yes		145 300 (98.2)	293 315 (98.0)	132 450 (98.0)	332 195 (98.1)	28.5

Abbreviation: SPEI, standardized precipitation evapotranspiration index.

^a^ Drought exposure for 27 521 participants was missing. SPEI-12 denotes the standardized precipitation evapotranspiration index at a 12-month timescale.

^b^ Prevalence was calculated based on the occurrences of intimate partner violence against women during the past 12 months before the survey.

**Figure 2.**
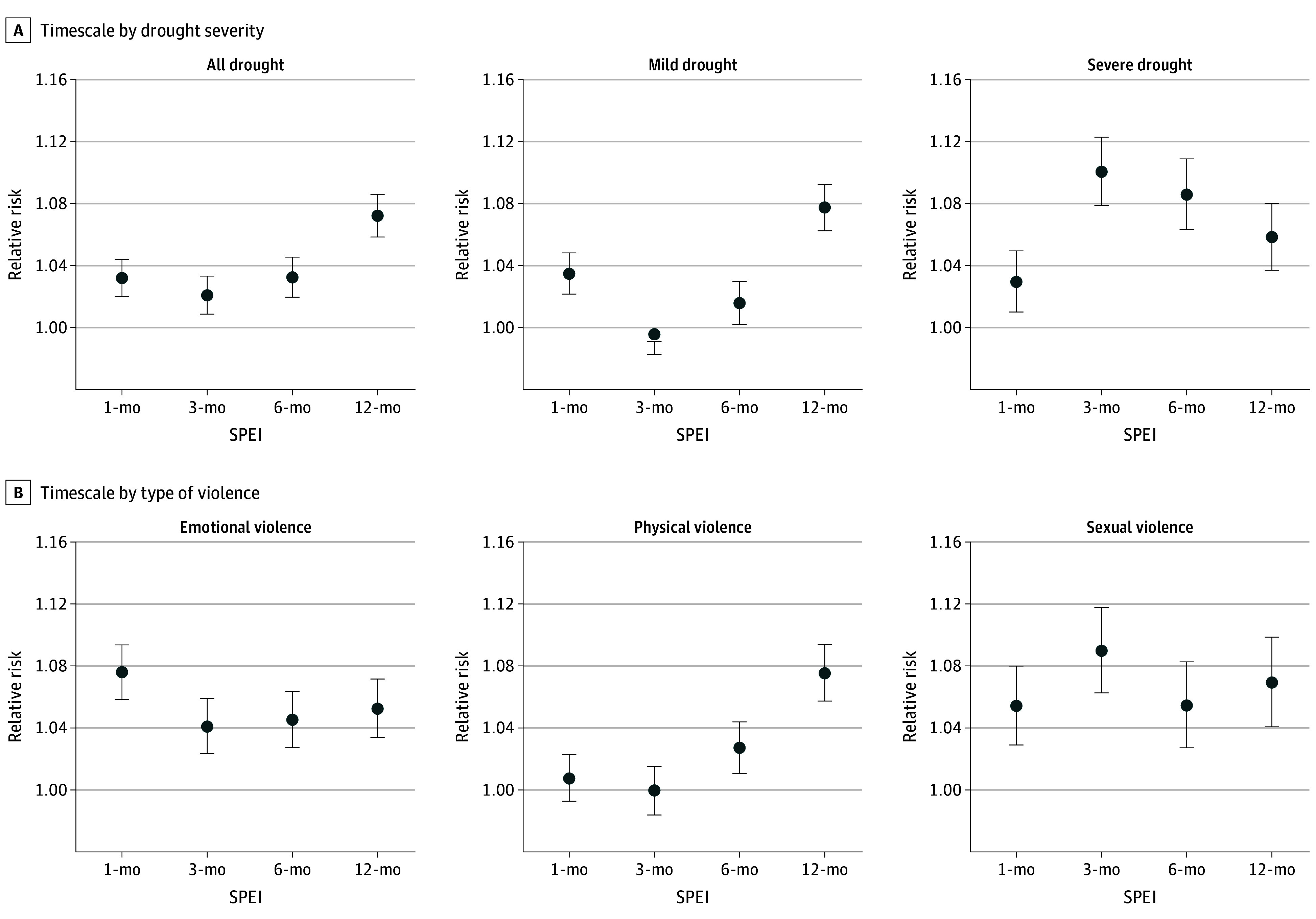
Associations Between Risk of Intimate Partner Violence and Exposure to Drought Represented by the Standardized Precipitation and Evapotranspiration Index (SPEI) at Various Timescales

Exposure to overall drought at any timescale was positively associated with the risk of IPV, with the highest RR at a timescale of 12 months (RR, 1.07; 95% CI, 1.06-1.09) (Figure 2A). Mild and severe drought manifested different patterns, with the association with mild drought also peaking at the 12-month scale (RR, 1.08; 95% CI, 1.06-1.09), whereas SPEI-3 severe drought showed the largest effect size for the association with IPV (RR, 1.10; 95% CI, 1.08-1.12) (Figure 2A). When analyzing different types of IPV separately, effect sizes were larger for associations estimated at a shorter timescale for emotional (SPEI-1) and sexual (SPEI-3) IPV. Conversely, we found a null association between physical violence and SPEI-1 or SPEI-3 drought, with the highest RR for SPEI-12 drought (Figure 2B).

The duration of the association with drought decreased with the increase in the timescale. Specifically, the positive and significant association of SPEI-1, SPEI-3, SPEI-6, and SPEI-12 drought persisted for 12, 9, 8, and 3 months, respectively (eFigure 5 in Supplement 1).

**Figure 3.**
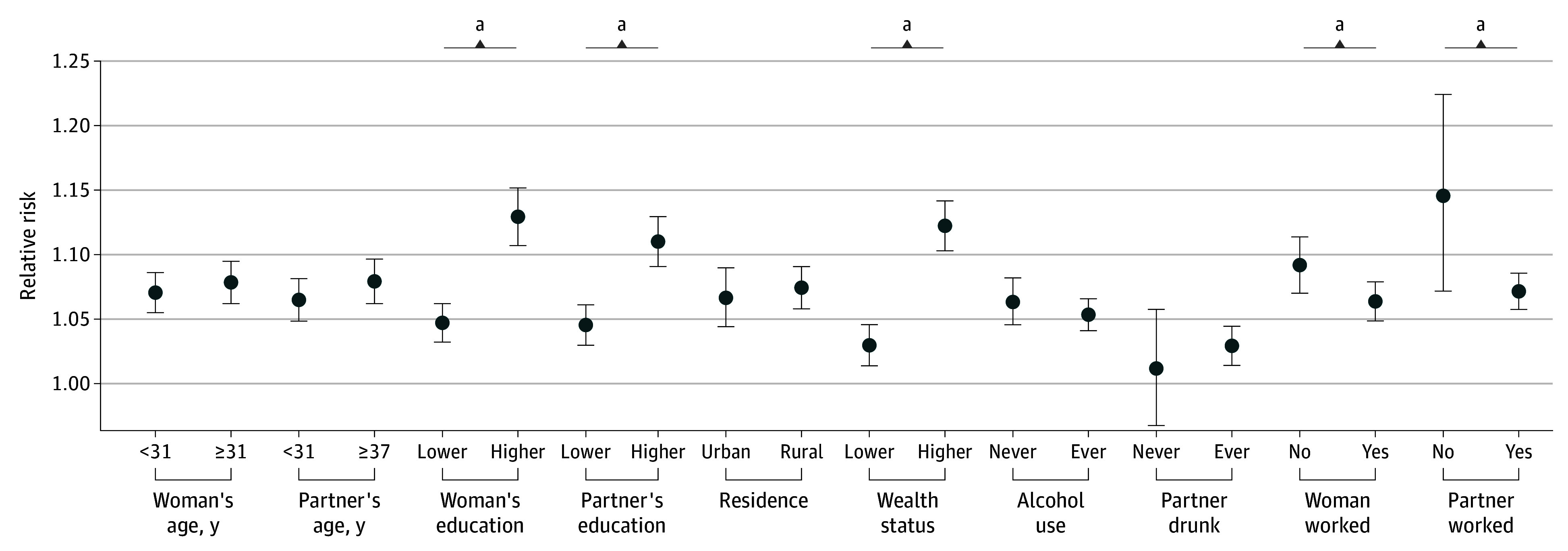
Associations Between Risk of Intimate Partner Violence and Exposure to Drought at a Timescale of 12 Months Stratified by Demographic, Socioeconomic, and Behavioral Characteristics ^a^Statistically significant pairwise differences (*P* < .05).

We performed the effect modification analysis and interaction test between drought and extreme heat only for SPEI-12 drought given its largest estimate in the main model. We noted statistically significantly larger RRs for the association among individuals with higher education, wealthier households, and employment in the year before the survey, compared with their counterparts with lower education, lower household wealth status, and unemployment status (Figure 3). We found no significant effect modification by other variables.

**Figure 4.**
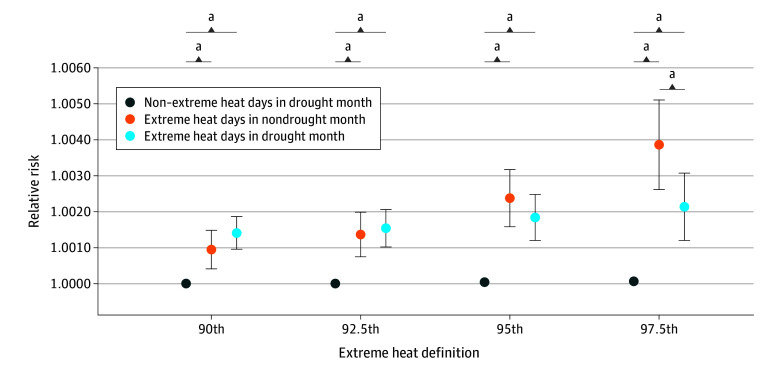
Interactive Associations Between Extreme Heat Days With Various Thresholds and Drought at a Timescale of 12 Months on Intimate Partner Violence ^a^Statistically significant pairwise differences (*P* < .05).

Year-round exposure to drought months and extreme heat days were both significantly and positively associated with a higher risk of IPV, with larger estimates for SPEI-1 drought and extreme heat with the strongest intensity (97.5th percentile) (eFigure 6 in Supplement 1). Surprisingly, we found that exposure to SPEI-12 drought significantly decreased the negative association of extreme heat with the highest intensity (97.5th percentile), and we did not observe a significant difference between extreme heat estimates for other extreme heat definitions (Figure 4). Exposure to extreme heat at various intensities in either nondrought months or drought months substantially increased the risk, compared with nonexposure to extreme heat (Figure 4). Different types of calculated RERI gradually decreased with the increase in extreme heat threshold (eTable 7 in Supplement 1). We also found a negative and significant additive interaction between SPEI-12 drought and extreme heat with the 97.5th percentile as the threshold (eTable 7 in Supplement 1).

Our estimate was found to be robust, except for a nonmeaningful increase in the association with total rainfall adjusted. The application of sampling weight also slightly inflated the estimate with a logistic regression model structure (eTable 8 in Supplement 1).

## Discussion

This cross-sectional study found a positive and consistent association between IPV and drought exposure at various timescales in LMICs, with the association estimated to persist for months. Furthermore, drought alleviated the negative impact of extreme heat with the highest intensity.

Previous evidence on the association between drought and IPV has been based on a simple indicator of water supply deficiencies such as limited precipitation.^17,18,19,20,21,22^ This study furthers our knowledge of the influence of drought, a complex multifaceted climate condition, considering multiple timescales and meteorological conditions on the risk of common types of IPV. We revealed a positive association not only for medium- and long-term drought (SPEI-6 and SPEI-12), coinciding with previous studies in Africa, Asia, and Latin America,^17,18,19^ but also for short-term drought at a timescale of 1 or 3 months, although the mechanism of varying associations by drought severity and by IPV type needs further investigation.

Our understanding of the potential pathways through which drought influences IPV remains quite limited. Agricultural deterioration, such as crop failure and livestock loss due to drought, and the subsequent household financial crisis, could introduce family primary income earners (more likely to be men in LMICs) to a series of mental disorders, such as poverty-related stress, anxiety, and depression,^29^ increasing the chance of IPV. Trauma exposure among natural disaster survivors can also potentially exacerbate IPV risks.^13^ The financial dependence on men already causes compromised empowerment of women, and drought conditions could further worsen their autonomy through reduced female employment and income, potentially enhancing men’s marital control.^19^ Furthermore, the unemployment status also increases the time women spend with their abusive partners. In addition, the deteriorating societal environment further weakens protection for women, and therefore enables a higher possibility of gender-based violence.^12^

To our knowledge, this study was the first to discover a time lag in the association between drought and a health outcome. The extended periods show potentially indirect pathways of drought exposure affecting IPV. Additionally, shorter lags at longer timescales indicate a greater imperative for governmental and social alleviative interventions to be implemented during severe and persistent droughts, mitigating the impact of the drought on IPV. In contrast, shorter drought periods might not prompt the same level of response or awareness, potentially leading to a latent adverse effect. This differentiation highlights the importance of timely interventions to address IPV risks associated with varying drought durations. The mechanism behind the unexpectedly larger effect sizes for associations among more educated individuals and wealthier households remain unclear. However, more educated individuals are more likely to report any form of IPV, while others will only report the most extreme cases of violence. Moreover, it is possible that men who were better educated or provided more financial support to their families manifested increased tendencies toward controlling behaviors and experienced heightened financial strain induced by poverty exacerbated by drought. The reproducibility of the effect modification by these baseline characteristics needs further investigation.

There is minimal evidence of the joint impact of drought and extreme heat on human health. A 2024 study^30^ also employed RERI and estimated a negative compounding effect of drought and heatwave on child mental health outcomes. We found a similar interactive effect for extreme heat with the strongest intensity. The underlying mechanism is unclear. As extreme heat intensified, we observed a declining difference in the associations with extreme heat days in drought and nondrought months. This indicates a weakening modifying effect of drought on extreme heat and an increasingly predominant role of extreme heat in their combined impact as temperatures approach the high-end extreme. With the increase in compound drought and extreme heat events in a warming world,^31^ their interactive effect on health warrants further exploration given the anticipated characteristic shift in frequency, intensity, and duration. To our knowledge, this is the first study exploring the compound influence of drought and extreme heat on IPV, 2 extreme events made more frequent by climate change. The negative interaction we estimated indicated a more effective intervention targeting IPV if we mitigate these 2 environmental hazards sequentially rather than jointly. More studies are warranted to examine the interactive effect of extreme heat with various thresholds.

### Limitations

Several limitations should be acknowledged. First, despite the use of the most strict criteria to ensure temporality, we were unable to conduct a more precise exposure assessment due to the unknown timing of IPV occurrence during the 12-month period. Second, IPV was self-reported through interviews and thus is susceptible to inaccurate recall, causing outcome misclassification. Third, the unequal temporal scales of drought (in months) and extreme heat (in days) hindered us from defining compound days in which drought and extreme heat co-occurred at a finer scale. Fourth, we stratified the analysis by 10 variables and tested the interaction by various drought and extreme heat variables, which may increase the likelihood of chance association due to multiple comparisons. We consider the effect modification and interaction analysis as exploratory and in need of further investigation. Fifth, our study was also prone to exposure misclassification (whether individuals were actually exposed to drought or extreme heat) because data on adaptive measures and individual mobility were unavailable.

## Conclusions

In conclusion, we estimated an increased risk of different types of IPV associated with drought conditions with various severities and durations. With foreseeable mounting individual and concurrent extreme events due to climate change,^32^ more effective efforts, such as emotional regulation through intervention, should be undertaken to prevent domestic violence.

## Notes

Supplement 1. **eAppendix 1.** Model Specification of Lagged Association Evaluation **eAppendix 2.** Calculation of Relative Excess Risk Due to Interaction (RERI) **eAppendix 3.** Sensitivity Analysis Specification **eTable 1.** Definitions of Different Types of Intimate Partner Violence **eTable 2.** Included Countries, Survey Years, and Number of Participants **eTable 3.** Descriptive Statistics for the Number of Overall Drought Months and Number of Extreme Heat Days During the 23rd to 12th Months Before the Survey **eTable 4.** Number of Women (Among All 494 471 Women) Exposed to Overall Drought at Various Timescales or Extreme Heat With Various Definitions **eTable 5.** Number of Women (Among All 494 471 Women) Exposed to Extreme Heat With Various Definitions by Exposure to Overall Drought at a Timescale of 1 or 3 Months During the 23rd to 12th Months Before the Survey **eTable 6.** Number of Women (Among All 494 471 Women) Exposed to Extreme Heat With Various Definitions by Exposure to Overall Drought at a Timescale of 6 or 12 Months During the 23rd to 12th Months Before the Survey **eTable 7.** Relative Excess Risk Due to Interaction Between Overall Drought and Extreme Heat **eTable 8.** Comparison Between Multiple Sensitivity Analyses and Main Analysis Using Overall Drought at a Timescale of 12 Months as the Exposure Variable **eFigure 1.** Timeframes to Calculate the Drought Indicator (SPEI) at a Timescale of 12 Months in the 12th Month Before the Survey and Extreme Heat Indicator During the 23rd to 12th Months Before the Survey **eFigure 2.** Global Prevalence for Emotional, Physical, and Sexual Intimate Partner Violence (Emotional Violence Data in Bangladesh and Colombia Were Missing) **eFigure 3.** Country-Specific Distributions of the Number of Drought Months (Overall Drought, Including Mild and Severe Drought) Across All 9-km Grids, Sorted by Area-Weighted Mean Value (Diamonds) During 2002-2019 **eFigure 4.** Distribution of Total Number of Drought Months at Different Timescales Across All 9-km Grids During 2002–2019 **eFigure 5.** Lagged Associations Between Overall Drought at Different Timescales and Any Intimate Partner Violence **eFigure 6.** The Association Between Intimate Partner Violence and Number of Drought Months by Timescale and Number of Extreme Heat Days by Threshold During the 23rd to 12th Months Before the Survey

Supplement 2.
Data Sharing Statement
