# Supplementary material for: Drought, Extreme Heat, and Intimate Partner Violence in Low- and Middle-Income Countries
Source: JAMA Netw Open. 2025 Aug 20;8(8):e2527818. doi: 10.1001/jamanetworkopen.2025.27818 (PMC12368687; doi:10.1001/jamanetworkopen.2025.27818)
Supplement: Supplement 1. — eAppendix 1. Model Specification of Lagged Association Evaluation eAppendix 2. Calculation of Relative Excess Risk Due to Interaction (RERI) eAppendix 3. Sensitivity Analysis Specification eTable 1. Definitions of Different Types of Intimate Partner Violence eTable 2. Included Countries, Survey Years, and Number of Participants eTable 3. Descriptive Statistics for the Number of Overall Drought Months and Number of Extreme Heat Days During the 23rd to 12th Months Before the Survey eTable 4. Number of Women (Among All 494 471 Women) Exposed to Overall Drought at Various Timescales or Extreme Heat With Various Definitions eTable 5. Number of Women (Among All 494 471 Women) Exposed to Extreme Heat With Various Definitions by Exposure to Overall Drought at a Timescale of 1 or 3 Months During the 23rd to 12th Months Before the Survey eTable 6. Number of Women (Among All 494 471 Women) Exposed to Extreme Heat With Various Definitions by Exposure to Overall Drought at a Timescale of 6 or 12 Months During the 23rd to 12th Months Before the Survey eTable 7. Relative Excess Risk Due to Interaction Between Overall Drought and Extreme Heat eTable 8. Comparison Between Multiple Sensitivity Analyses and Main Analysis Using Overall Drought at a Timescale of 12 Months as the Exposure Variable eFigure 1. Timeframes to Calculate the Drought Indicator (SPEI) at a Timescale of 12 Months in the 12th Month Before the Survey and Extreme Heat Indicator During the 23rd to 12th Months Before the Survey eFigure 2. Global Prevalence for Emotional, Physical, and Sexual Intimate Partner Violence (Emotional Violence Data in Bangladesh and Colombia Were Missing) eFigure 3. Country-Specific Distributions of the Number of Drought Months (Overall Drought, Including Mild and Severe Drought) Across All 9-km Grids, Sorted by Area-Weighted Mean Value (Diamonds) During 2002-2019 eFigure 4. Distribution of Total Number of Drought Months at Different Timescales Across All 9-km Grids During 2002–2019 eFigure [file jamanetwopen-e2527818-s001.pdf]

Supplemental Online Content

Wang P, Chu L, Ban J, et al. Drought, extreme heat, and intimate partner violence in low- and middle-income countries. *JAMA Netw Open.* 2025;8(8):e2527818. doi:10.1001/jamanetworkopen.2025.27818

eAppendix 1. Model Specification of Lagged Association Evaluation

eAppendix 2. Calculation of Relative Excess Risk Due to Interaction (RERI)

eAppendix 3. Sensitivity Analysis Specification

eTable 1. Definitions of Different Types of Intimate Partner Violence

eTable 2. Included Countries, Survey Years, and Number of Participants

eTable 3. Descriptive Statistics for the Number of Overall Drought Months and Number of Extreme Heat Days During the 23rd to 12th Months Before the Survey

eTable 4. Number of Women (Among All 494 471 Women) Exposed to Overall Drought at Various Timescales or Extreme Heat With Various Definitions

eTable 5. Number of Women (Among All 494 471 Women) Exposed to Extreme Heat With Various Definitions by Exposure to Overall Drought at a Timescale of 1 or 3 Months During the 23rd to 12th Months Before the Survey

eTable 6. Number of Women (Among All 494 471 Women) Exposed to Extreme Heat With Various Definitions by Exposure to Overall Drought at a Timescale of 6 or 12 Months During the 23rd to 12th Months Before the Survey

eTable 7. Relative Excess Risk Due to Interaction Between Overall Drought and Extreme Heat

eTable 8. Comparison Between Multiple Sensitivity Analyses and Main Analysis Using Overall Drought at a Timescale of 12 Months as the Exposure Variable

eFigure 1. Timeframes to Calculate the Drought Indicator (SPEI) at a Timescale of 12 Months in the 12th Month Before the Survey and Extreme Heat Indicator During the 23rd to 12th Months Before the Survey

eFigure 2. Global Prevalence for Emotional, Physical, and Sexual Intimate Partner Violence (Emotional Violence Data in Bangladesh and Colombia Were Missing)

© 2025 Wang P et al. *JAMA Network Open*.

eFigure 3. Country-Specific Distributions of the Number of Drought Months (Overall Drought, Including Mild and Severe Drought) Across All 9-km Grids, Sorted by Area-Weighted Mean Value (Diamonds) During 2002-2019

eFigure 4. Distribution of Total Number of Drought Months at Different Timescales Across All 9-km grids during 2002–2019

eFigure 5. Lagged Associations Between Overall Drought at Different Timescales and Any Intimate Partner Violence

eFigure 6. The Association Between Intimate Partner Violence and Number of Drought Months by Timescale and Number of Extreme Heat Days by Threshold During the 23rd to 12th Months Before the Survey

This supplemental material has been provided by the authors to give readers additional information about their work.

© 2025 Wang P et al. *JAMA Network Open*.

eMethods 1. Model specification of lagged association evaluation

Log-binomial generalized additive models with distributed lag non-linear models were used to investigate the potential lag of association between drought and intimate partner violence (IPV). The equation is as follows:

$$Log(P_i) = \alpha + cb(drought_i, lag = 11) + ns(temperature_i, df = 3) + ns(year_i, df = 3) + \beta COV_i + \gamma \varphi_i$$

where P_i is the probability of experiencing IPV; *cb*() is the cross-basis function simultaneously accounting for exposure-response and lag-response relationship for the drought indicator at different timescales, with a linear function for the former and a natural cubic spline (*ns*) function with three degrees of freedom for the latter. We used 11 months as the maximum lag period to model its potential delayed effect during an entire year (lag 0–11) after the exposure. COV_i is a matrix of baseline characteristics, including man's age, man's education, and wealth index. φ_i is a composite indicator of country and area of residence (urban or rural). The same random effect term as in the main analysis (i.e., nested country and survey cluster) was not used due to model nonconvergence. Only any IPV (emotional, physical, and sexual IPV combined) and overall drought (mild and severe drought combined) were examined in this lagged association analysis.

© 2025 Wang P et al. *JAMA Network Open*.

eMethods 2. Calculation of relative excess risk due to interaction (RERI)

We calculated RERI in two ways, using the following formula:

$$RERI_{RR} = RR_{11} - RR_{10} - RR_{01} + 1$$

Then we can ascertain positive additive interaction if RERI>0 and negative additive interaction if RERI<0.[1]

First, we counted the total number of overall drought days (number of drought months × 30) at a timescale of 12 months and the total number of extreme heat (EH) days above various thresholds (90th/92.5th/95th/97.5th percentile of the survey-cluster-specific distribution of maximum temperature during 2002–2019) during the 23rd–12th months before the survey. Then, we tested their interaction using the model as in the main analysis, excluding the temperature variable (due to the strong correlation between temperature and EH variables), and the formula above. We calculated an empirical 95% confidence interval for each RERI by generating 1000 bootstrap samples.

Second, we separately computed the total number of EH days in drought months, EH days in non-drought months, and non-EH days in drought months during the aforementioned 12-month period, and included these metrics in the same model as in the main analysis, excluding the temperature variable (consistent with the first method). The RRs in the formula above were directly extracted from the models. Specifically, RR_{11} is the coefficient for the number of EH days in drought months, RR_{01} is the coefficient for the number of EH days in non-drought months, and RR_{10} is the coefficient for the number of non-EH days in drought months. The variances of RERIs were calculated following the methodology described by Li et al.[2] Then the 95% confidence intervals of RERIs were calculated as $RERI \pm 1.96 \times \sqrt{Var(RERI)}$.

© 2025 Wang P et al. *JAMA Network Open*.

eMethods 3. Sensitivity analysis specification

We performed various sensitivity analyses to test model robustness. First, we adjusted for the following variables in respective models: 1) the number of EH days above the 90th percentile in the 12th month before the survey; 2) a natural spline of average monthly total rainfall with three degrees of freedom, calculated during the same calculation period for the standardized precipitation evapotranspiration index; 3) the month of the survey; 4) woman's age at first marriage; and 5) gross domestic product per capita[3] for each survey cluster during the survey year (2015 data were used for surveys conducted after 2015). Second, we removed the average mean temperature from the main model. Finally, we applied sampling weights from the DHS to take into account the complex survey design. However, generalized linear mixed logistic regression models were used instead since the log-binomial link does not accommodate sampling weights with decimals.

© 2025 Wang P et al. *JAMA Network Open*.

eTable 1. Definitions of different types of intimate partner violence

Intimate partner violence	Definition
Emotional violence	Ever humiliated
	Ever threatened with harm
	Ever insulted or made feel bad
Physical violence	Ever pushed, shaken, or had something thrown by
	Ever slapped
	Ever punched with fist or something harmful
	Ever kicked or dragged
	Ever tried to be strangled or burned
	Ever threatened with knife, gun, or other weapons
	Ever attacked with knife, gun, or other weapons
	The arm was ever twisted or the hair was ever pulled
Sexual violence	Ever physically forced sex when not wanted
	Ever forced other sexual acts with not wanted

© 2025 Wang P et al. *JAMA Network Open*.

eTable 2. Included countries, survey years, and number of participants

Country	Survey year(s)	Participants	Country	Survey year(s)	Participants
Angola	2015–2016	7669	Liberia	2006–2007; 2019–2020	6244
Bangladesh	2007	4467	Malawi	2010; 2015–2016	10,778
Benin	2017–2018	4488	Mali	2006; 2012–2013; 2018	15,398
Burkina Faso	2010	10,003	Mozambique	2011	5824
Burundi	2016–2017	7366	Myanmar	2015–2016	3425
Cambodia	2005–2006; 2014	5791	Namibia	2013	1448
Cameroon	2011; 2018–2019	8694	Nepal	2011; 2016–2017	7331
Chad	2014–2015	3811	Nigeria	2008; 2013; 2018	50,411
Colombia	2005–2006; 2014	34,681	Pakistan	2017–2018	4085
Comoros	2012	2529	Peru	2003–2009	36,699
Democratic Republic of the Congo	2007; 2013–2014	8540	Philippines	2008; 2017	21,693
Côte d'Ivoire	2011–2012	5006	Rwanda	2005; 2010–2011; 2014–2015; 2019–2020	7978
Dominican Republic	2007; 2013	15,367	Senegal	2017–2019	5634
Ethiopia	2016	4720	Sierra Leone	2013; 2019	8362
Gabon	2012	4133	South Africa	2016	4003
Gambia	2019–2020	1953	Tanzania	2009–2010; 2015–2016	13,286
Ghana	2008	1835	Timor-Leste	2009–2010; 2016	5856
Haiti	2005–2006; 2012; 2016–2017	13,639	Togo	2013–2014	5374
Honduras	2011–2012	12,494	Uganda	2006; 2011; 2016	10,985
India	2015–2016	66,013	Zambia	2007; 2013–2014; 2018–2019	20,997
Kenya	2008–2009; 2014	9417	Zimbabwe	2005–2006; 2010–2011; 2015	16,044

© 2025 Wang P et al. *JAMA Network Open*.

eTable 3. Descriptive statistics for the number of overall drought months and number of extreme heat days during the 23rd–12th months before the survey

	Mean (SD)	Minimum	Median	Maximum	Interquartile range
**Drought months**					
SPEI-1	4.0 (2.0)	0	4	11	3–5
SPEI-3	4.1 (2.7)	0	4	12	2–6
SPEI-6	4.1 (3.4)	0	4	12	1–6
SPEI-12	4.1 (4.4)	0	2	12	0–8
**Extreme heat days**					
≥90th percentile	35.8 (17.2)	0	34	155	25–44
≥92.5th percentile	27.0 (15.2)	0	25	144	18–34
≥95th percentile	18.2 (12.5)	0	16	128	10–23
≥97.5th percentile	9.2 (8.6)	0	8	99	4–12

© 2025 Wang P et al. *JAMA Network Open*.

eTable 4. Number of women (among all 494,471 women) exposed to overall drought at various timescales or extreme heat with various definitions. SPEI: standardized precipitation evapotranspiration index.

	Missing exposure	Exposed		Non-exposed	
		Cases	Non-cases	Cases	Non-cases
Overall drought in the 12th month before the survey					
SPEI-1	27,566	47,550	117,951	85,927	215,477
SPEI-3	27,565	44,305	112,586	89,178	220,837
SPEI-6	27,541	41,641	109,139	91,845	224,305
SPEI-12	27,521	44,018	111,333	89,474	222,125
Overall drought during the 23rd–12th months before the survey					
SPEI-1	27,521	130,949	327,151	2543	6307
SPEI-3	27,521	121,517	307,127	11,975	26,331
SPEI-6	27,521	107,032	273,728	26,460	59,730
SPEI-12	27,521	85,174	219,008	48,318	114,450
Extreme heat during the 23rd–12th months before the survey					
≥90th percentile	27,521	132,653	331,883	839	1575
≥92.5th percentile	27,521	131,649	329,920	1843	3538
≥95th percentile	27,521	129,587	326,002	3905	7456
≥97.5th percentile	27,521	121,696	309,102	11,796	24,356

© 2025 Wang P et al. *JAMA Network Open*.

eTable 5. Number of women (among all 494,471 women†) exposed to extreme heat with various definitions by exposure to overall drought at a timescale of 1 or 3 months during the 23rd–12th months before the survey. SPEI: standardized precipitation evapotranspiration index.

	**Exposed to extreme heat**		**Non-exposed to extreme heat**	
	**Cases**	**Non-cases**	**Cases**	**Non-cases**
Exposed to SPEI-1				
≥90th percentile	130,177	325,722	772	1429
≥92.5th percentile	129,290	323,972	1659	3179
≥95th percentile	127,315	320,255	3634	6896
≥97.5th percentile	119,707	304,105	11,242	23,046
Non-exposed to SPEI-1				
≥90th percentile	2476	6161	67	146
≥92.5th percentile	2359	5948	184	359
≥95th percentile	2272	5747	271	560
≥97.5th percentile	1989	4997	554	1310
Exposed to SPEI-3				
≥90th percentile	121,199	306,464	318	663
≥92.5th percentile	120,795	305,594	722	1533
≥95th percentile	119,182	302,486	2335	4641
≥97.5th percentile	112,406	287,950	9111	19,177
Non-exposed to SPEI-3				
≥90th percentile	11,454	25,419	521	912
≥92.5th percentile	10,854	24,326	1121	2005
≥95th percentile	10,405	23,516	1570	2815
≥97.5th percentile	9290	21,152	2685	5179

† A total of 27,521 women missed exposure to extreme heat or exposure to overall drought during the 23rd–12th months before the survey.

© 2025 Wang P et al. *JAMA Network Open*.

eTable 6. Number of women (among all 494,471 women[†]) exposed to extreme heat with various definitions by exposure to overall drought at a timescale of 6 or 12 months during the 23rd–12th months before the survey. SPEI: standardized precipitation evapotranspiration index.

	Exposed to extreme heat		Non-exposed to extreme heat	
	Cases	Non-cases	Cases	Non-cases
Exposed to SPEI-6				
≥90th percentile	106,834	273,205	198	523
≥92.5th percentile	106,677	272,782	355	946
≥95th percentile	105,798	270,860	1234	2868
≥97.5th percentile	100,692	259,650	6340	14,078
Non-exposed to SPEI-6				
≥90th percentile	25,819	58,678	641	1052
≥92.5th percentile	24,972	57,138	1488	2592
≥95th percentile	23,789	55,142	2671	4588
≥97.5th percentile	21,004	49,452	5456	10,278
Exposed to SPEI-12				
≥90th percentile	85,107	218,802	67	206
≥92.5th percentile	85,000	218,479	174	529
≥95th percentile	84,387	217,106	787	1902
≥97.5th percentile	81,046	209,960	4128	9048
Non-exposed to SPEI-12				
≥90th percentile	47,546	113,081	772	1369
≥92.5th percentile	46,649	111,441	1669	3009
≥95th percentile	45,200	108,896	3118	5554
≥97.5th percentile	40,650	99,142	7668	15,308

[†] A total of 27,521 women missed exposure to extreme heat or exposure to overall drought during the 23rd–12th months before the survey.

© 2025 Wang P et al. *JAMA Network Open*.

eTable 7. Relative excess risk due to interaction between overall drought at a timescale of 12 months and extreme heat. SPEI: standardized precipitation evapotranspiration index.

	**Extreme heat definitions**			
	**≥90th**	**≥92.5th**	**≥95th**	**≥97.5th**
Method 1	2.77e-06 (-9.13e-08, 6.29e-06)	1.41e-06 (-2.56e-06, 4.46e-06)	-2.35e-06† (-8.65e-06, -3.11e-07)	-8.93e-06† (-1.94e-05, -7.67e-06)
Method 2	4.77e-04 (-9.00e-05, 1.04e-03)	1.70e-04 (-5.13e-04, 8.53e-04)	-5.78e-04 (-1.47e-03, 3.15e-04)	-1.78e-03† (-3.21e-03, -3.56e-04)

† Statistically significant results. All 95% confidence intervals are noted in the parentheses. Method 1 represents the method involving the interaction term between drought and extreme heat in the model. Method 2 represents the method incorporating the total number of EH days in drought months, EH days in non-drought months, and non-EH days in drought months in the same model.

© 2025 Wang P et al. *JAMA Network Open*.

eTable 8. Comparison between multiple sensitivity analyses and main analysis using overall drought at a timescale of 12 months as the exposure variable. The lower and upper bounds denote the 95% confidence interval.

Model family	Model setting	Estimate	Lower bound	Upper bound
Log-binomial generalized linear mixed model	Main analysis	1.072	1.058	1.086
	Extreme heat day adjusted	1.071	1.058	1.085
	Total rainfall adjusted	1.082	1.068	1.096
	Survey month adjusted	1.073	1.060	1.087
	Age at first marriage adjusted	1.075	1.061	1.089
	GDP per capita adjusted	1.071	1.057	1.085
	Mean temperature removed	1.071	1.058	1.085
Generalized linear mixed logistic regression model	No sampling weights	1.123	1.099	1.148
	Sampling weights adjusted	1.145	1.118	1.174

© 2025 Wang P et al. *JAMA Network Open*.

eFigure 1. Timeframes to calculate the drought indicator (SPEI) at a timescale of 12 months in the 12th month before the survey (A) and extreme heat indicator during the 23rd–12th months before the survey (B). SPEI: standardized precipitation evapotranspiration index.

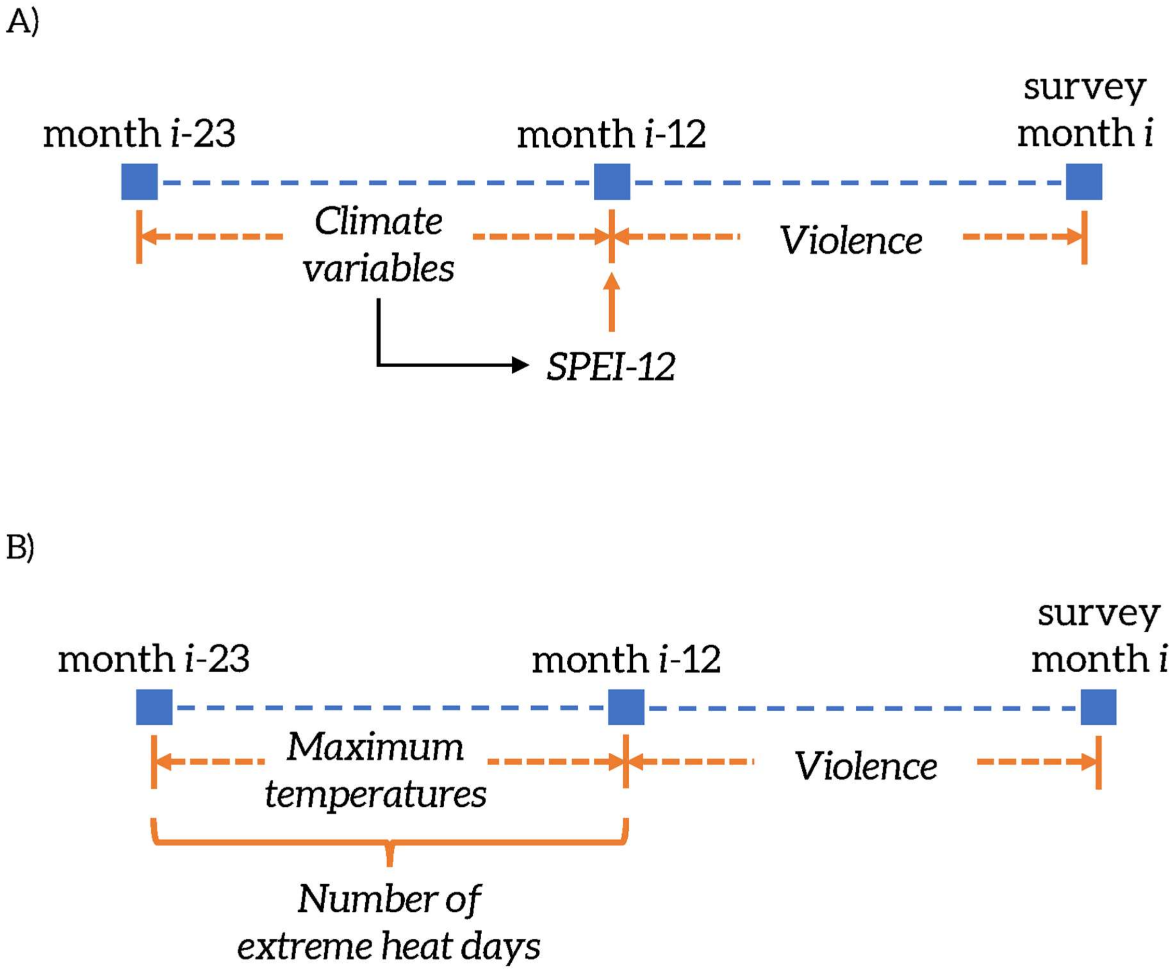

© 2025 Wang P et al. *JAMA Network Open*.

eFigure 2. Global prevalence for emotional, physical, and sexual intimate partner violence (emotional violence data in Bangladesh and Colombia were missing)

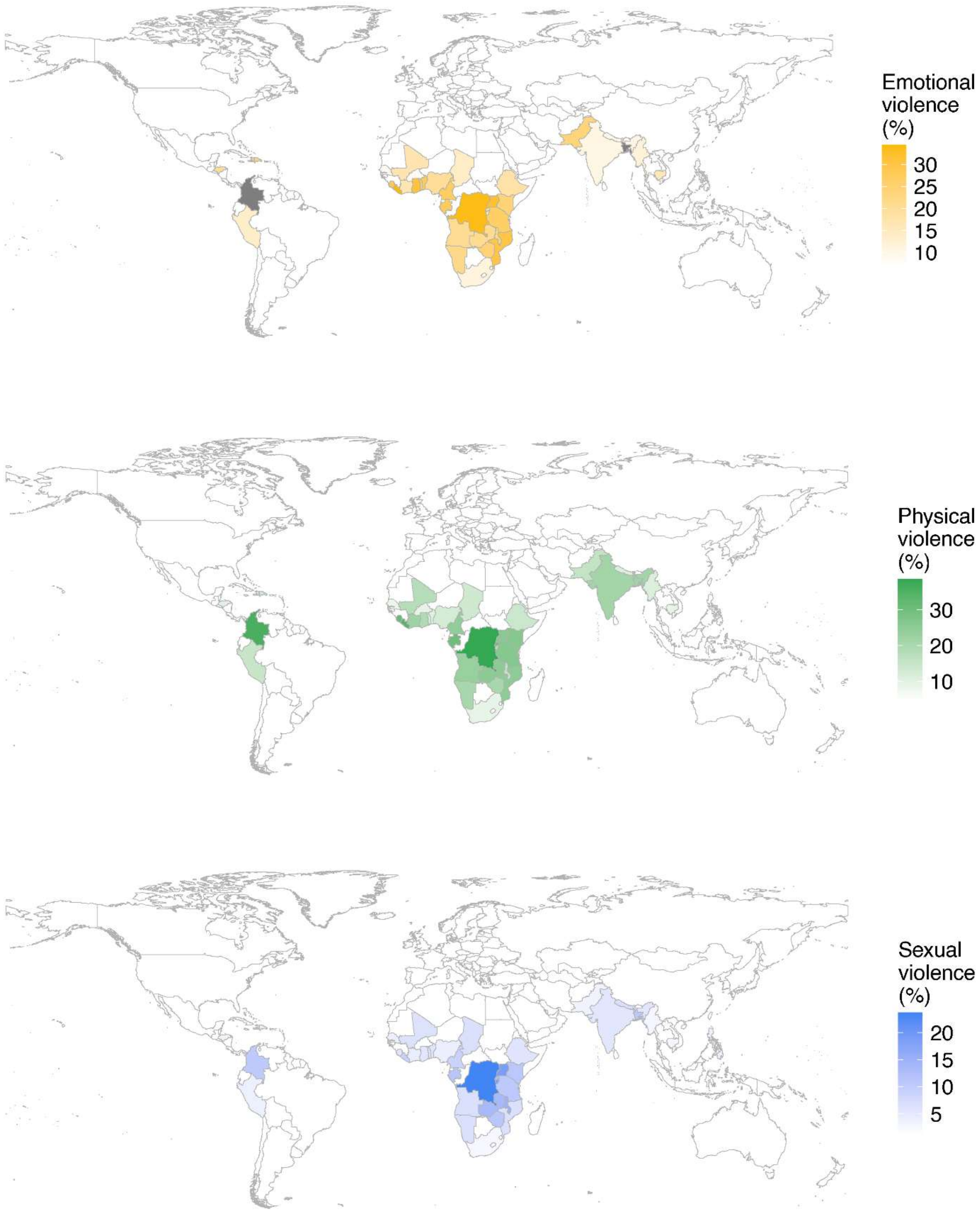

© 2025 Wang P et al. *JAMA Network Open*.

eFigure 3. Country-specific distributions of the number of drought months (overall drought, including mild and severe drought) across all 9-km grids, sorted by area-weighted mean value (diamonds) during 2002–2019. SPEI: standardized precipitation evapotranspiration index.

© 2025 Wang P et al. *JAMA Network Open*.

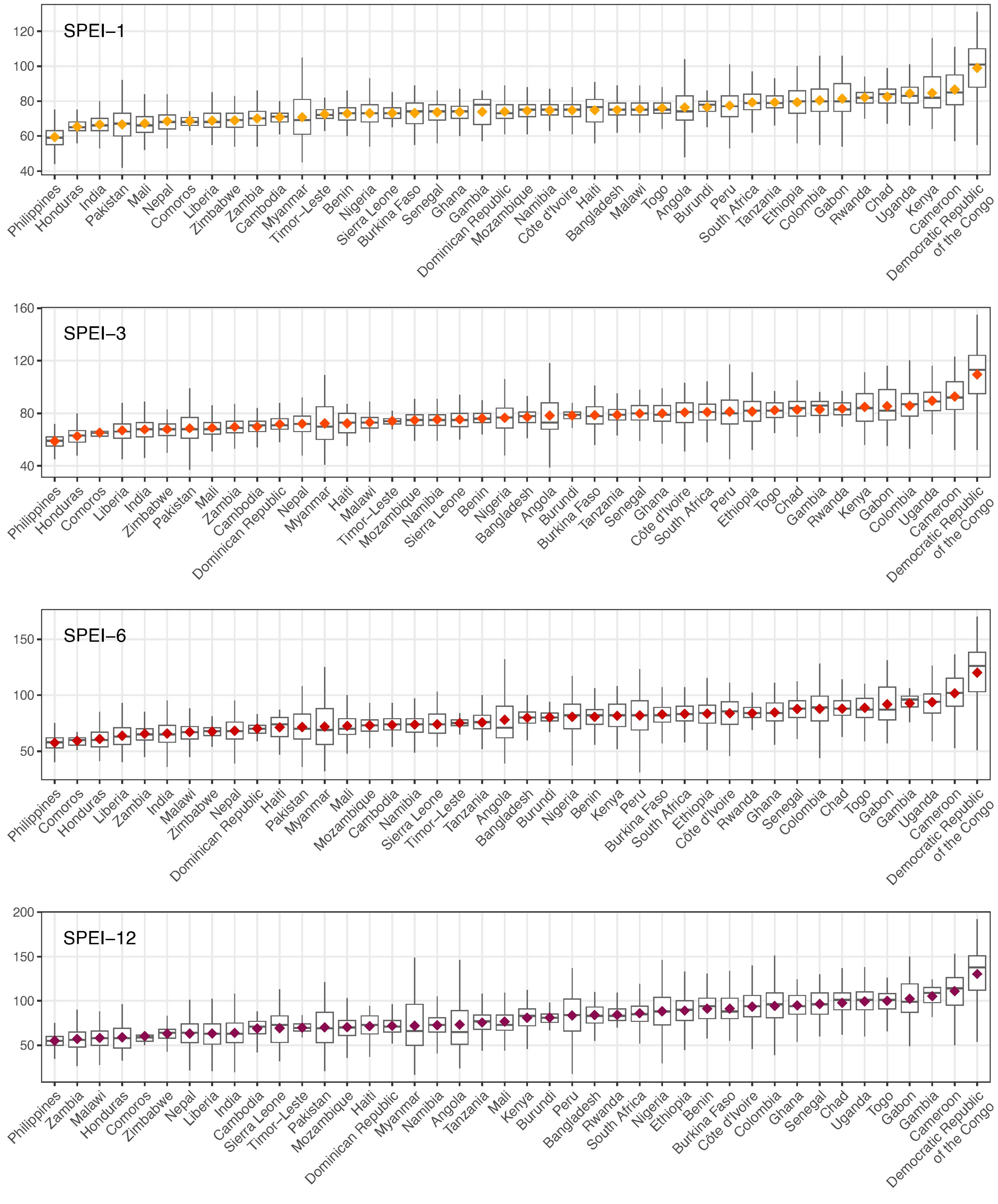

© 2025 Wang P et al. *JAMA Network Open*.

eFigure 4. Distribution of total number of drought months at different timescales across all 9-km grids during 2002–2019. The discrepancy in the time period is because the drought indicator was calculated for the 12th month preceding the survey. The raincloud plots include the median values (center lines), third and first quartiles (box limits), and 1.5x the interquartile range (whiskers).

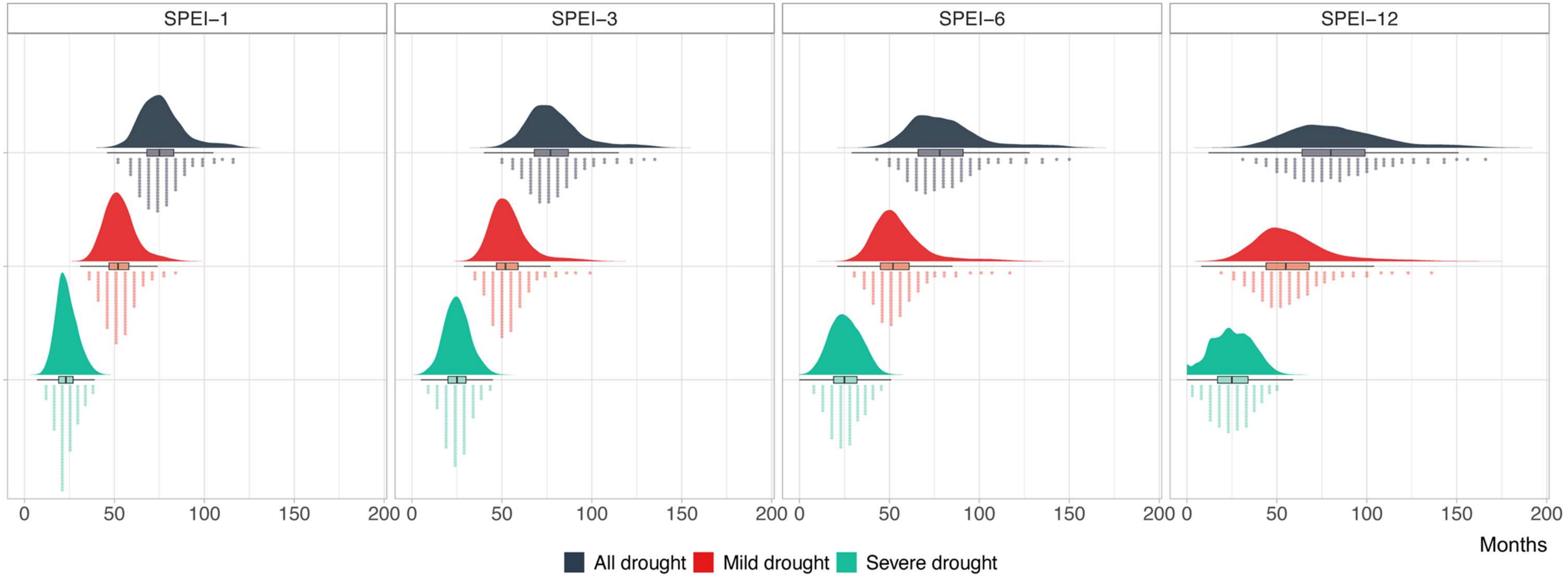

© 2025 Wang P et al. *JAMA Network Open*.

eFigure 5. Lagged associations between overall drought at different timescales and any intimate partner violence. SPEI: standardized precipitation evapotranspiration index.

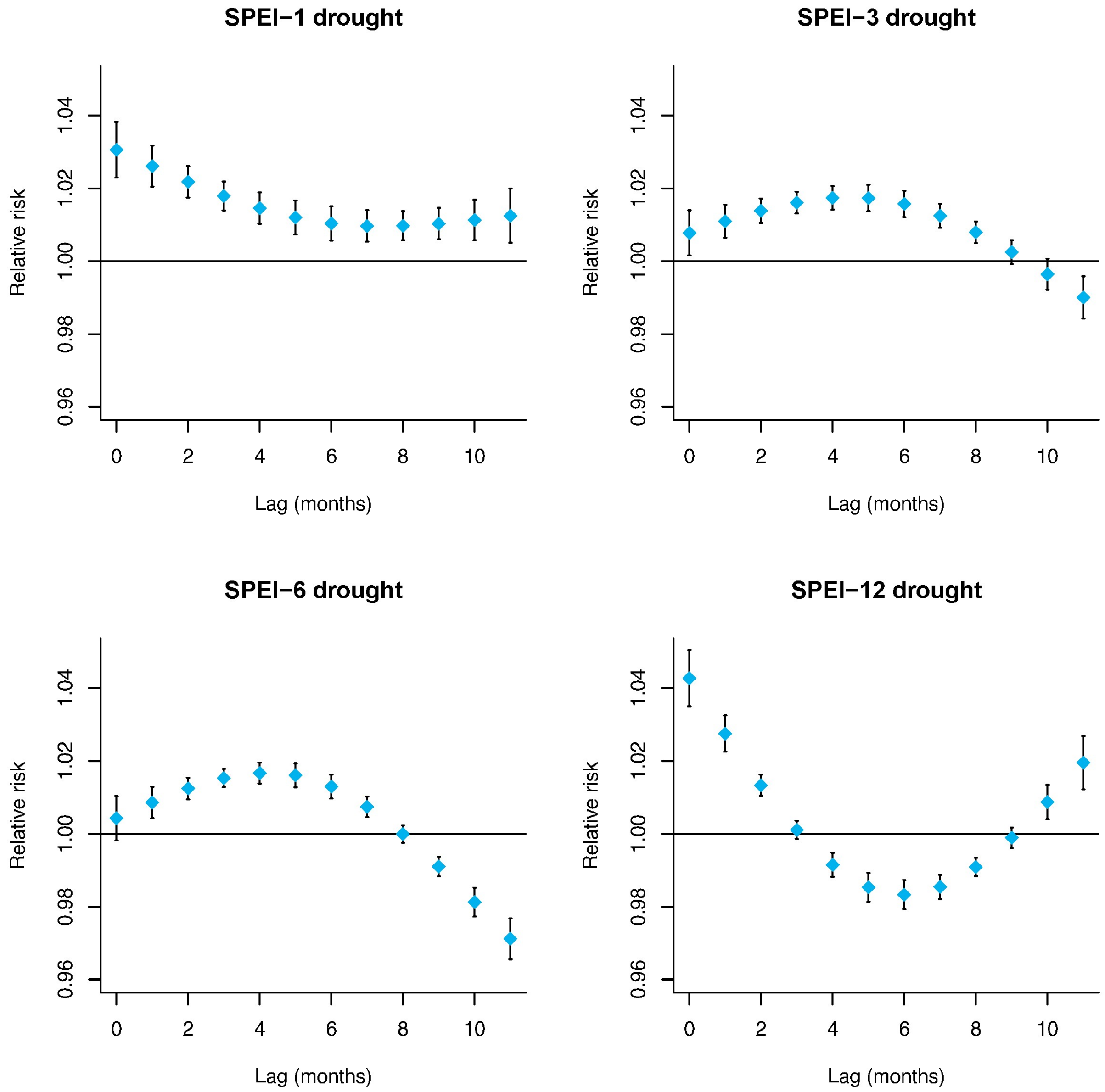

© 2025 Wang P et al. *JAMA Network Open*.

eFigure 6. The association between intimate partner violence and number of drought months by timescale and number of extreme heat days by threshold during the 23rd–12th months before the survey. The red and orange bars represent mean estimates and the two bars around those represent 95% confidence interval. SPEI: standardized precipitation evapotranspiration index.

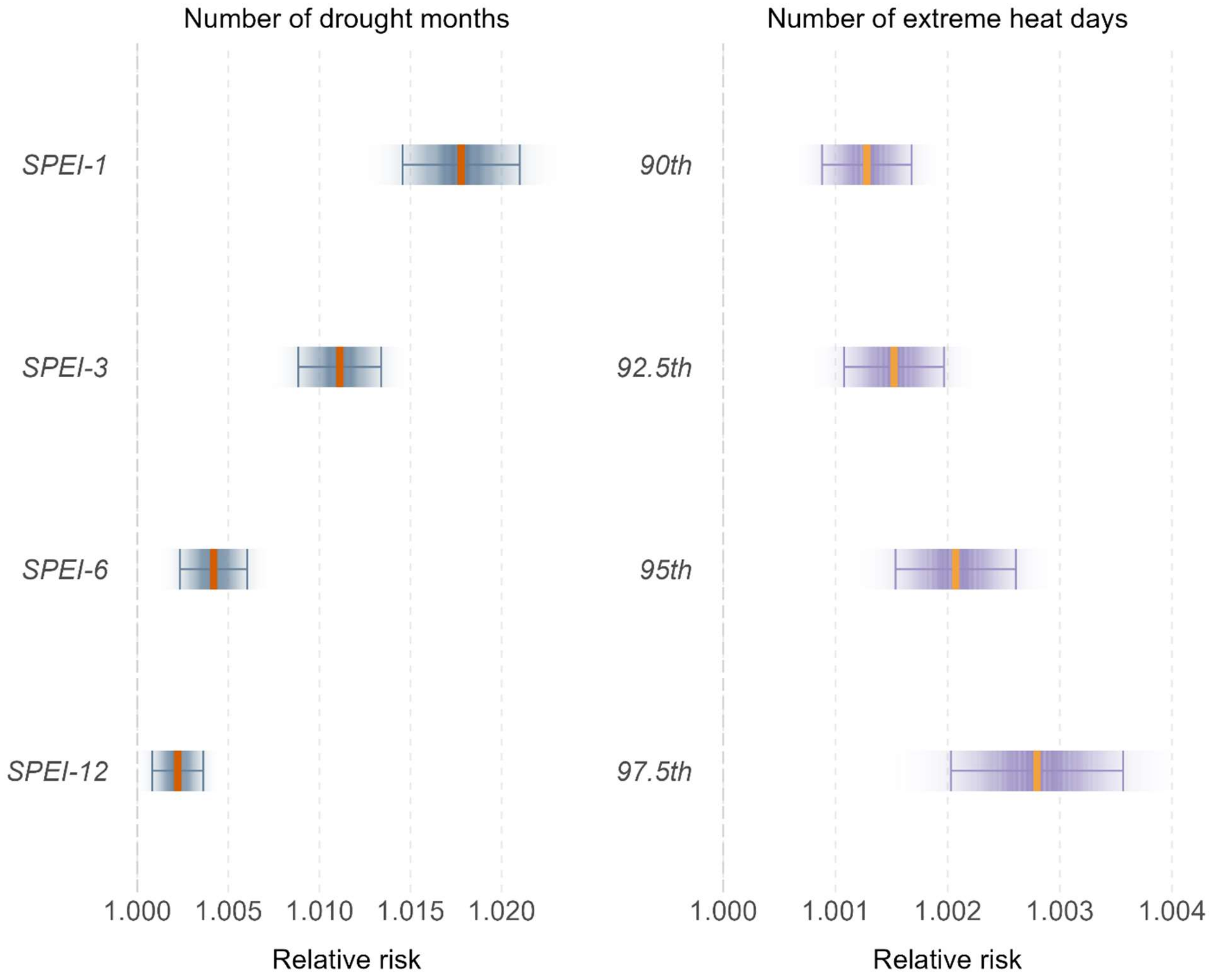

© 2025 Wang P et al. *JAMA Network Open*.

Reference:

1. VanderWeele TJ, Knol MJ. A tutorial on interaction. *Epidemiol Methods* 2014; **3**(1): 33-72.
2. Li R, Chambless L. Test for additive interaction in proportional hazards models. *Ann Epidemiol* 2007; **17**(3): 227-36.
3. Kummu M, Taka M, Guillaume JHA. Data Descriptor: Gridded global datasets for Gross Domestic Product and Human Development Index over 1990-2015. *Scientific Data* 2018; **5**.

© 2025 Wang P et al. *JAMA Network Open*.
