# Supplementary material for: Drought, Extreme Heat, and Intimate Partner Violence in Low- and Middle-Income Countries
Source: JAMA Netw Open. 2025 Aug 20;8(8):e2527818. doi: 10.1001/jamanetworkopen.2025.27818 (PMC12368687; doi:10.1001/jamanetworkopen.2025.27818)
Supplement: Supplement 2. — Data Sharing Statement [file jamanetwopen-e2527818-s002.pdf]

Data Sharing Statement

Wang. Drought, Extreme Heat, and Intimate Partner Violence in Low- and Middle-Income Countries. *JAMA Netw Open*. Published August 20, 2025. doi:10.1001/jamanetworkopen.2025.27818

Data
Data available: No

Additional Information
Explanation for why data not available: Data on intimate partner violence and socioeconomic status in this study were collected from the Demographic and Health Surveys program (https://dhsprogram.com/), which is freely available upon request. Publicly available meteorological records were obtained from the fifth generation ECMWF atmospheric reanalysis of the global climate (ERA5-Land, https://cds.climate.copernicus.eu/).
